# The Complement-Fixation Test in the Diagnosis of Gonorrheal Infections of the Genito-Urinary Tract in the Male and Female

**Published:** 1913-03

**Authors:** B. F. Stout

**Affiliations:** San Antonio, Texas


### The Complement-Fixation Test in the Diagnosis of Gonorrheal Infections of the Genito-Urinary Tract in the Male and Female.

B. F. STOUT, M. D., SAN ANTONIO, TEXAS.

The complement-fixation test in the diagnosis of gonorrheal infections is not so well known as the complement-fixation test in the diagnosis of syphilis, known as the Wassermann reaction; but, from extensive experiments and studies made on the subject, it promises to be of hardly less importance.

Without going into the history of the literature to date, I may say that Schwartz and McNeil, of New York, have done the practical work that has placed the reaction in the group of useful, practical laboratory tests. Their first work was published May, 1911, in *American Journal Medical Science*, while their most recent publication appeared in the same journal in December, 1912.

A number of articles have appeared in other journals during this time, fully corroborating the work done by the above men. My own experience with this test, while not large, has been most satisfactory.

The test therefore rests upon a foundation of a large experience, in which the findings of various investigators fully agree.

In regard to technique, it may be said that this is in principle the same as the Wassermann reaction, substituting the gonorrheal antigen for the syphilitic.

It appears that about twelve different strains of gonococci are found in this country; therefore, it is necessary to use a polyvalent antigen containing all these strains. Now as to the value of the reaction in general work, first considering under what conditions the reaction is present or absent. Schwartz and McNeil believe:

1. That a positive reaction means a focus of living gonococci in the body.

2. That a negative reaction, while not excluding the presence of gonococci, has a greater negative value than the negative Wassermann.

A positive reaction is not to be expected earlier than the fourth week of the disease. Cases in which the disease is limited to the anterior urethra do not show the reaction, while in the female some involvement of the cervix is necessary for the anti-bodies to develop. However, cases in which the disease remains limited to the urethra are rare and unimportant, while the same may be said of female infections.

These same authors believe, further, that if a negative reaction is obtained seven or eight weeks after clinical cure that the case may be looked on as cured.

All who have had much experience in the microscopic search for the gonococcus in chronic urethral or cervical discharges will agree that it is a most uncertain and unsatisfactory method. Especially is this true of the female genito-urinary tract. Bacteriological assistance is only of value in acute cases.

It is not necessary to point out the great assistance this test will prove to be in detecting this condition in pelvic disorders of women. In the male, its chief value will be in the determination of the presence or absence of living gonococci in chronic cases or in joint or kidney involvement. It has been long recognized that it is almost impossible to say whether a man is cured or not. And this question, which comes up before the doctor many hundred times in the cases who present themselves to find if they are safe to marry, will now have an answer.

While a negative reaction is not absolute proof of cure, still it is very strong evidence, and will be of the greatest value in these cases. Many cases that would otherwise be regarded as cured will be found still active and, therefore, dangerous.

The following figures will speak for themselves:

1. In cases regarded as post-gonorrheal a positive reaction is obtained in 31 per cent.

2. In sixty-two cases of chronic prostatitis, giving a history of infection within three years, a positive reaction was obtained in 54.8 per cent.

3. In 165 cases, looked upon as clinically cured for three months or more, a positive reaction was obtained in 13.2 per cent.

In conclusion, let me say that no man should marry who has had gonorrhea, unless he gives not only evidence of clinical cure, and negative bacteriological findings, but also a negative complement-fixation test. In this way the number of the innocently infected will be reduced to very small figures.

---

When Kocher's method fails to reduce a recent dislocation of the shoulder, it is usually because the surgeon has proceeded too rapidly. Deliberately is the only way to work quickly.—*American Journal of Surgery.*

It is becoming more and more evident from the expressions of the lay press in all parts of the United States that public opinion will not only allow but would eagerly welcome the sterilization of the feeble-minded, the criminal and those afflicted with inheritable diseases. The fact, recently given widespread circulation through the press, that there are over 300,000 feeble-minded persons in the institutions of the United States—and many more at large—has struck the lay editorial imagination forcibly, and editorials laying bare the conditions and approving the method of sterilization as the only remedial measure at our command are appearing in all parts of the country. The time is ripe for those States which have not yet taken action to follow in the lead of those who have. It is perfectly true in the case of the feeble-minded and the criminal that the animal instincts are highly developed and that, if the individual were forbidden marriage, irregular more or less permanent connections or even more or less promiscuity would result. The feeble-minded should be given institutional care, but sterilized as well to prevent accidents; the criminals should be sterilized because the major part of them are at large during most of their lives; and bearers of such as yet uncontrolled disease as leprosy should be sterilized and permitted to marry, as otherwise the procreation of children who are almost certain to be leprous will continue despite the absence of ceremonial unions.

As yet, apparently, there has been no protest against sterilization and the propaganda of eugenics has fortunately had ample time to commend itself to the general common sense. Presently it can, of course, be expected that anti-vaccination and anti-vivisection societies—encouragers of disease, and therefore of poverty and crime—will take up a position of opposition to this beneficent measure, and pass resolutions concerning the liberty of the individual and send petitions to Legislatures. But all of this brings us back to the subject of education—and it should by this time be borne in upon the minds of the medical profession that the education of the public in matters medical is one of the most crying needs of the hour, for it is the public that makes the laws. Therefore, look to the curricula of the public schools and the columns of the popular press.—*Lancet-Clinic.*

---

If blood is vomited in large quantity it is important to distinguish, by the history and physical signs, between gastric ulcer and ruptured varicosities of the esophagus.—*American Journal of Surgery.*

